# Experience-Dependent Counselor-Client Brain Synchronization during Psychological Counseling

**DOI:** 10.1523/ENEURO.0236-20.2020

**Published:** 2020-09-22

**Authors:** Ya Zhang, Tian Meng, Yaxi Yang, Yi Hu

**Affiliations:** School of Psychology and Cognitive Science, Shanghai Changning-ECNU Mental Health Center, East China Normal University, Shanghai, China

**Keywords:** counselor-client brain synchronization, experienced psychotherapists, fNIRS hyperscanning, psychotherapy experience, temporo-parietal junction, working alliance

## Abstract

The role of the counselor’s experience in building an alliance with the clients remains controversial. Recently, the expanding nascent studies on interpersonal brain synchronization (IBS) using functional near-infrared spectroscopy (fNIRS) on human subjects have hinted at the possible neural substrates underlying the relationship qualities between the counselor-client dyads. Our study assessed the clients’ self-report working alliance (WA) as well as simultaneously measured IBS by fNIRS in 14 experienced versus 16 novice counselor-client dyads during the first integrative-orientation psychological counseling session. We observed that synchronous brain activity patterns were elicited from the right temporo-parietal junction (rTPJ) across counselor-client dyads. Furthermore, such IBS, together with alliance quality, was especially evident when counselors had more psychotherapy experience. Time-lagged counselor-client brain synchronization might co-vary with the alliance (goal component) when the client’s brain activity preceded that of the counselor. These findings favor the notion that the IBS between counselor-client associated with the WA is an experience-dependent phenomenon, suggesting that a potential adaptive mechanism is embedded in psychological counseling.

## Significance Statement

Recent expanding nascent studies on interpersonal brain synchronization (IBS) during the interpersonal communication process using functional near-infrared spectroscopy (fNIRS) have hinted at the possible neural substrates underlying the effective relationship/alliance between the counselor-client dyads. By using fNIRs, our study found that the experienced counselors could build better alliance and stronger IBS of the right temporo-parietal junction (rTPJ) with the clients versus novice counselors, at least in the first session. This result supports the notion that a counselor’s level of experience is important in establishing positive alliance and the increased IBS of the rTPJ in the experienced counselor group versus the novice counselor group might indicate the neural basis of the better alliance during the psychological counseling process.

## Introduction

Effective relationship or working alliance (WA) might be the most common and essential therapeutic factor in the field of contemporary clinical psychology (Wampold and Imel, 2015). Some researchers have emphasized that the establishment of an effective relationship is the most important criterium for measuring expertise in psychological counseling (Constantino et al., 2011; Hill et al., 2017a). Just as Ackerman and Hilsenroth (2003) concluded in their review, the number of clinical experience years might contribute to higher client-rated and counselor-rated WA scores.

However, the mostly-cohesive body of literature on this subject suggests that experience does not significantly affect a counselor’s contribution to alliance qualities (for review, see Hersoug et al., 2001; Tschuschke et al., 2015; Dawson, 2018). Therefore, it remains unclear whether experienced counselors can build a better relationship or alliance with clients than novice counselors can. The contradictory results in the literature may be partly because of the use of self-reported scales (Mallinckrodt and Nelson, 1991; Tschuschke et al., 2015) or self-reported experiences (Oddli and Halvorsen, 2014) at a single point during treatment (typically early or late) under different psychotherapy approaches. However, the WA grew during the whole process of psychological counseling and might have different patterns of development (Kivlighan and Shaughnessy, 2000). Better alliance measures or the establishment of signals focusing on what exactly the counselor needs to do are needed (Hill et al., 2017b). Fortunately, recent expanding nascent studies on interpersonal brain synchronization (IBS) during the interpersonal communication process using functional near-infrared spectroscopy (fNIRS) have hinted at the possible neural substrates underlying the effective relationship/alliance between the counselor-client dyads.

In the past ten years, abundant evidence has suggested that the level of IBS correlates with the level of successful understanding/sharing between partners to enable mental coordination (Stephens et al., 2010; Mu et al., 2018). Neural synchrony was anchored in the moments of social gaze and positive affect, which was related to the degree of social connectedness among interacting partners (Kinreich et al., 2017). In fact, recent researchers found a general increase of IBS during cooperative and engaging interpersonal interaction, which suggested the vital contribution of IBS to successful communication (e.g., verbal communication, Jiang et al., 2012, 2015; semi-verbal communication, Osaka et al., 2015; nonverbal communication, Cui et al., 2012; Holper et al., 2012). In short, IBS might be influenced by interpersonal closeness/connectedness (Kinreich et al., 2017) and understanding/sharing (Stephens et al., 2010). Similarly, a recent study using fNIRS hyperscanning provided evidence that increased IBS within counselor-client dyads was associated with a better WA (Zhang et al., 2018) comparing with chatting dyads. These studies collectively suggest that IBS may be either a neural indicator or an objective measure of relationship or alliance qualities during the interpersonal interaction, which included the psychological counseling process.

In short, previous researches kept controversial about whether experienced counselors can build a better relationship or alliance with clients than novice counselors can. IBS during the interpersonal communication process provided possible neural indicators of effective alliance/relationship between counselor-client dyads. Accordingly, we aimed to compare IBS as well as the relationship qualities between the experienced counselor-client dyads and the novice counselor-client dyads by using fNIRS hyperscanning, which is a safe, non-invasive imaging modality (Ferrari and Quaresima, 2012) that uses NIR light projected to (source) and from (detractor) tissues to quantitatively monitor the levels of cortical oxyhemoglobin (oxyHb) and deoxyhemoglobin (deoxyHb). It can be used to investigate synchronous brain activities in natural unconstrained communication, which enabled the identification of IBS in the counselor-client dyads during psychological counseling.

Specifically, previous studies have demonstrated neuronal synchrony in the right temporo-parietal junction (rTPJ) during face-to-face interaction (Tang et al., 2016), or face-to-face psychological counseling (Zhang et al., 2018). The rTPJ plays an important role in establishing positive relationships, and may be associated with cognitive empathy (Atique et al., 2011) or social connectedness (Kinreich et al., 2017). Accordingly, we chose the rTPJ as the target region. In short, the current study compared the IBS of the rTPJ and WA between the experienced counselor-client dyads and the novice counselor-client dyads during the first-session psychological counseling.

## Materials and Methods

### Participants

Thirty right-handed college students undertook the client roles [all females; age range: 18–26 years; mean age (M_age_): 21.1 years] and were randomly assigned to either the novice-counselor or experienced-counselor group. The students were recruited from the college counseling centers where they had voluntarily applied to receive psychological counseling. All 30 clients had experienced moderate stress or developmental issues with academic activities, interpersonal relationships, or adaptation to college life and had no known psychiatric or physical conditions.

Previous studies found that synchronous brain activity during cooperative interpersonal activities depends on gender of partner (Cheng et al., 2015; Lu et al., 2020). In our study, we chose only female participants (female clients and female counselors in two groups) to avoid the gender effect on brain synchronization. For the novice-counselor group, we recruited five female psychological counselors (age range: 23–29 years; M_age_: 24.8 years). They were first-year graduate students with 15- to 23-h experience in providing psychological counseling. Each novice counselor provided three to four clients with psychological counseling so that the novice counselor-client dyads amounted to 16. For the experienced counselor group, we recruited three female licensed psychological counselors (age range: 29–45 years; M_age_: 34.7 years) and 600- to 4000-h experience in providing psychological counseling. Each experienced counselor provided four to five clients with psychological counseling so that the experienced counselor-client dyads amounted to 14. Statistical comparison of the experience between the two groups of counselors confirmed that the experienced counselors had a significantly greater amount of experience than that of the novice counselors (*t*_(6)_ = 3.71, *p *< 0.001). The M_age_ of the counselors in each group did not differ significantly [experienced, mean (M): 34.7, SD: 8.96; novice, M: 24.8, SD: 2.49, *t*_(6)_ = 2.43, *p *>* *0.05].

Moreover, the eight counselors were instructed to provide the same type of counseling-integrative orientation. They were trained using a college counseling program that instructed them to focus on the emotional states and reported troubles of the client, as well as the client’s expectation for pursuing counseling. This program emphasized on helping the counselors to integrate different orientations and provide a brief psychological service for college students. All participants provided written informed consent and received United States $14.50 for their efforts. The University Committee on Human Research Protections of a local university approved the study protocol (HR 187–2018).

### Experimental procedure

**Figure 1.**
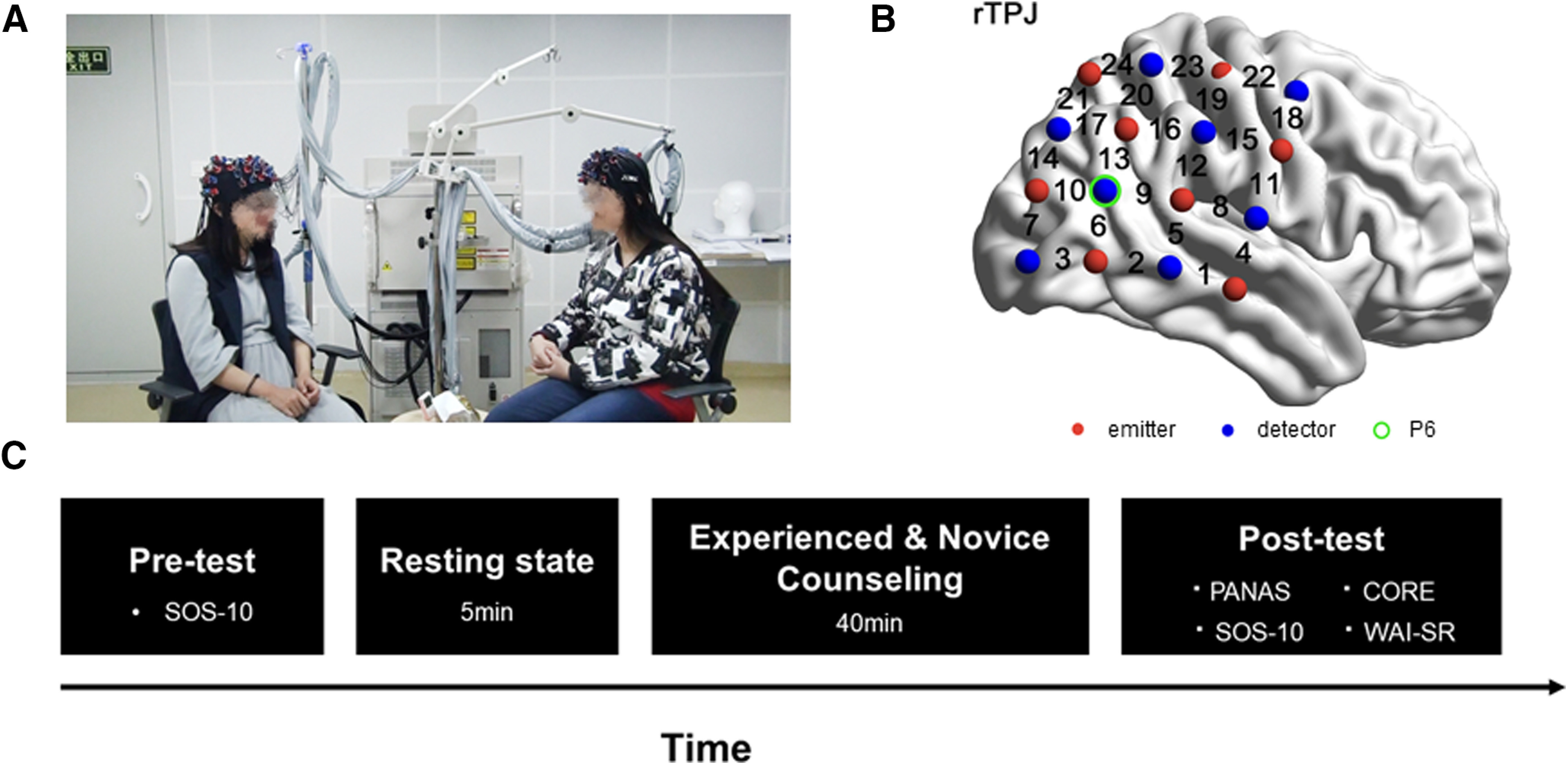
Experimental design. ***A***, Experimental set-up. ***B***, Experimental task and procedure. ***C***, The optode-probe set was placed over the rTPJ.

To emulate a natural counseling setting, each dyad was arranged so that the client (on the left) and counselor (on the right) sat at a 90° angle and a distance of 40 cm from each other (Fig. 1*A*). First, a 5-min initial resting-state was presented during the rest session (baseline), during the resting session, participants were asked to relax and sit comfortably without communication or eye contact; second, a 40-min psychological counseling period was presented as the task session, during which participants could observe nonverbal cues (e.g., gestures and facial expressions). During the whole task, the room was kept exclusively for the dyad, and the overall procedures were video recorded. The session ended on schedule when the research assistant knocked on the door and stopped the process.

### Subjective measurements and clinical assessment

Following the psychological counseling provided by either the novice or experienced counselors, participants were invited to complete the Chinese version of the Working Alliance Inventory-Short Revised (WAI-SR; Munder et al., 2010; Chinese version, Hsu et al., 2016) and the Chinese version (with permission of the author) of the Schwartz Outcome Scale-10 (SOS-10; Blais et al., 1999).

The WAI-SR included 12 items (Cronbach’s α = 0.82) that assessed three key aspects of the therapeutic WA (Bordin, 1979; Munder et al., 2010): goal, agreement about the client’s dissatisfaction (e.g., “The counselor and I collaborate on setting goals for my therapy”); task, a means of approaching the counseling (e.g., “I feel that the things I do in therapy will help me to accomplish the changes that I want”); bond, connection between counselors and clients (e.g., “I feel that my counselor appreciates me”). All items were rated on a 5-point Likert scale, ranging from 1 = never to 5 = always.

The SOS-10 was produced to measure the clinical improvement that occurred during routine psychological counseling. It included 10 items with a total score ranging from 0 to 60. Higher scores indicate greater psychological health and a better state of well-being. The SOS-10 was conducted before and after the psychological counseling process.

### NIRS data acquisition

An ETG-7100 optical topography system (Hitachi Medical Company) with customized optode probe sets was used to collect the fNIRS data. The absorption near-infrared light (wavelengths: 695 and 830 nm) was measured at a sampling rate of 10 Hz. Based on previous studies about the role of IBS in psychological counseling (Zhang et al., 2018), the rTPJ (Fig. 1*C*) was selected as the region of interest and a 4 × 4 probe set [eight emitters and eight detectors, forming 24 measurement channels (CHs)] was placed over the rTPJ regions referenced to P6 (10/20 international system; Fig. 1*B*). To determine the correspondence between the NIRS CHs and the measurement points on the cerebral cortex, the virtual registration method was used (Singh et al., 2005; Tsuzuki et al., 2007). Changes in oxyHb and deoxyHb were measured. Previous studies have shown that oxyHb concentration is a sensitive indicator of the change in rTPJ blood flow (Tang et al., 2016; Zhang et al., 2018); thus, our study focused solely on oxyHb concentration.

### Data analysis

#### Behavioral data

We compared WAI-SR scores between the two groups using two-sample *t* tests. The precounseling and postcounseling SOS-10 scores were used to evaluate clinical improvement. Statistical analyses were performed using SPSS software (version 22.0).

#### IBS

We collected and analyzed fNIRS data during the resting state and task sessions. After deleting the data corresponding to the first and last minute of the resting period, the remaining rest data were regarded as baseline. Considering that the first 5 min of the psychological counseling process mostly involved introducing the counseling frame and psychological counselor, there was little focus on topics or the client’s emotional state during that period. Hence, the data corresponding to the first 5 min of the 40-min psychological counseling period was deleted; the rest were retained as task-related data (lasting 35 min).

Considering that fNIRS might record global and cortical blood oxygen level-dependent (BOLD) activities, we used the principal component spatial filter algorithm (PCA; Zhang et al., 2016) to remove the global components. Thereafter, wavelet transform coherence (WTC) was used to estimate IBS between the clients and counselors; the analysis process was conducted in accordance with previous studies (Grinsted et al., 2004; Nozawa et al., 2016). This approach has been successfully applied in hyperscanning studies to detect synchronous brain activity between two individuals (Pan et al., 2017, 2018).

To identify the IBS increases that were specifically associated with psychological counseling, we performed the following steps. First, to identify the frequency ranges that were specifically associated with counseling, the data from the two groups were combined, and the IBS during the baseline stage was subtracted to obtain the task-related IBS. We then conducted one sample *t* test on the time-averaged task-related IBS from both groups, along with the full frequency range (0.01–0.1 Hz; according to previous fNIRS-based hyperscanning studies; Jiang et al., 2012). A threshold of *p *<* *0.0005 was applied to the results according to the method reported by Zheng et al. (2018). No further correction for multiple comparisons was applied because this analysis was only used to identify the pattern along the frequency range, rather than to obtain final results. Only the frequencies range (0.04–0.03 Hz) had CH combinations whose *p* value survived the thresholding.

Second, we selected the frequencies that were around the target frequency range (0.04–0.03 Hz) as well as their *p* values were <0.05. Then we obtained the extensive frequency range from 0.02 to 0.05 Hz. The coherence values within this frequency range were averaged. Afterward, we conducted one sample *t* test again on task-related IBS within the selected frequencies. Results were corrected with the false discovery rate (FDR) method for all CHs at *p *<* *0.05 level. We found that the selected CHs were those that detected the significantly-increased values in the two groups during the task-related frequencies. Finally, a series of *t* tests were conducted to determine any differences between the two groups in the IBS values for the selected CHs. Additionally, in our experienced counselor group, three counselors had different clinical experiences varying from 600 to 4000 h. To exclude the possibility that the IBS might differ across the experienced counselors, we performed one-way ANOVA with counselor (counselor 1 vs counselor 2 vs counselor 3) on the IBS and WA in experienced counselors’ group and found no significant effect involving counselors (all *p*s > 0.18).

In addition, to examine whether and when the counselor could predict the client’s state, we added various time lags to the computation of IBS increases (Stephens et al., 2010; Liu et al., 2017). The time course of the counselor’s brain activity was shifted forward relative to that of the client’s brain activity by −10–10 s (step = 2 s) and the IBS increases were recomputed and statistically tested. Data analysis was conducted again as mentioned above. Based on the different time lags, 10 different conditions (−2, −4, −6, −8, −10, 2, 4, 6, 8, and 10 s) were considered, and IBS data were recalculated using WTC under the ten conditions. Next, the time-averaged IBS along the task-related frequency range during the rest and task periods were recalculated, and task-related IBS was obtained by subtracting the IBS during the rest period from that during the task period. Finally, a two-sample *t* test was conducted to compare task-related IBS between the two groups and determine any differences across the different time-lagged conditions. The results were corrected for all CHs with *p *<* *0.05 across all time-lagged conditions using the FDR method.

Regarding clinical assessment, a 2 × 2 (group: novice counselor vs experienced counselor × pre-post: precounseling vs postcounseling) mixed ANOVA was conducted, with the group as the between-subject factor on SOS scores.

#### Neural-behavioral correlation

We chose only the CHs with (1) significant increased IBS comparing with the baseline and (2) significant IBS differences in two groups. Pearson correlational analyses were performed to explore the relationships among IBS, time-lagged IBS, and WA on different CHs in two groups separately.

## Results

### Behavioral results

**Figure 2.**
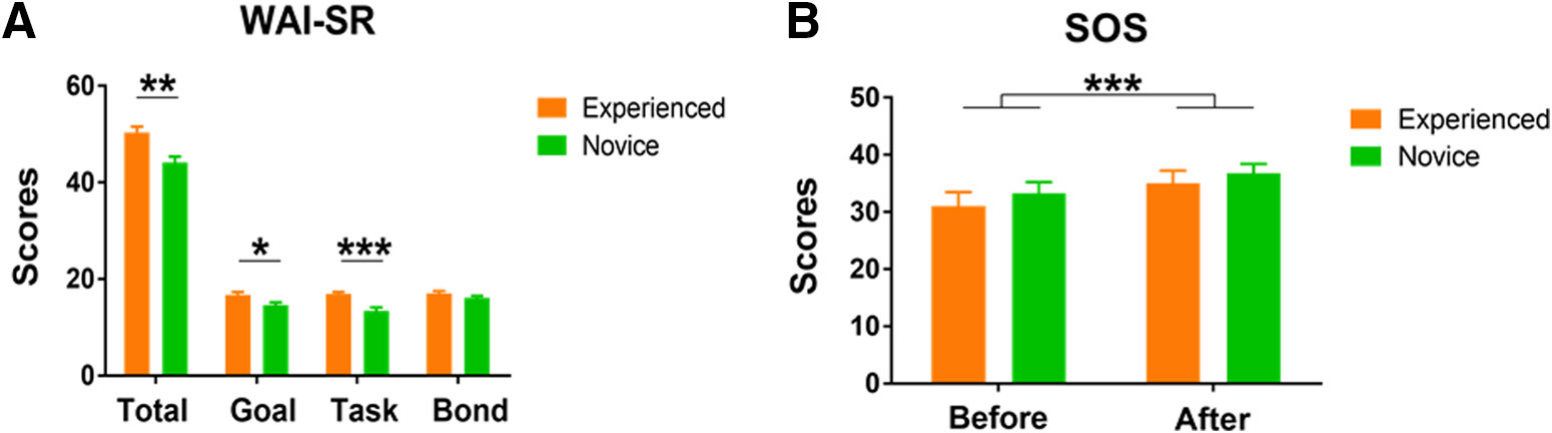
Behavioral results. ***A***, The total and individual dimension scores for the WAI-SR in the two groups. ***B***, The total scores of the SOS-10 before and after 40-min psychological counseling sessions in the two groups; *p <* *0.05, **p <* *0.01, ***p <* *0.001. Error bars indicate SEs.

The total scores and two subscales of the Chinese version of the WAI-SR were significantly higher in the experienced counselor group than in the novice counselor group [total scores: *t*_(28)_ =* *3.77, *p *=* *0.001; task (subscale): *t*_(28)_ =* *4.28, *p *<* *0.001; goal (subscale): *t*_(28)_ =* *2.45, *p *=* *0.021; Fig. 2*A*]. However, the subscale for the bond did not differ significantly between groups (*t*_(28)_* *=* *1.35, *p *=* *0.19).

The ANOVA results for clinical assessment indicated that the main effect of pre-post reached significance (*F*_(1,28)_ = 17.16, *p *<* *0.001; M_pre_ = 32.50, SD =* *8.42; M_post_ = 36.20, SD = 7.30). There was no significant interaction between the two factors (*F*_(1,28)_ = 0.85, *p *=* *0.37; Fig. 2*B*).

### IBS

**Figure 3.**
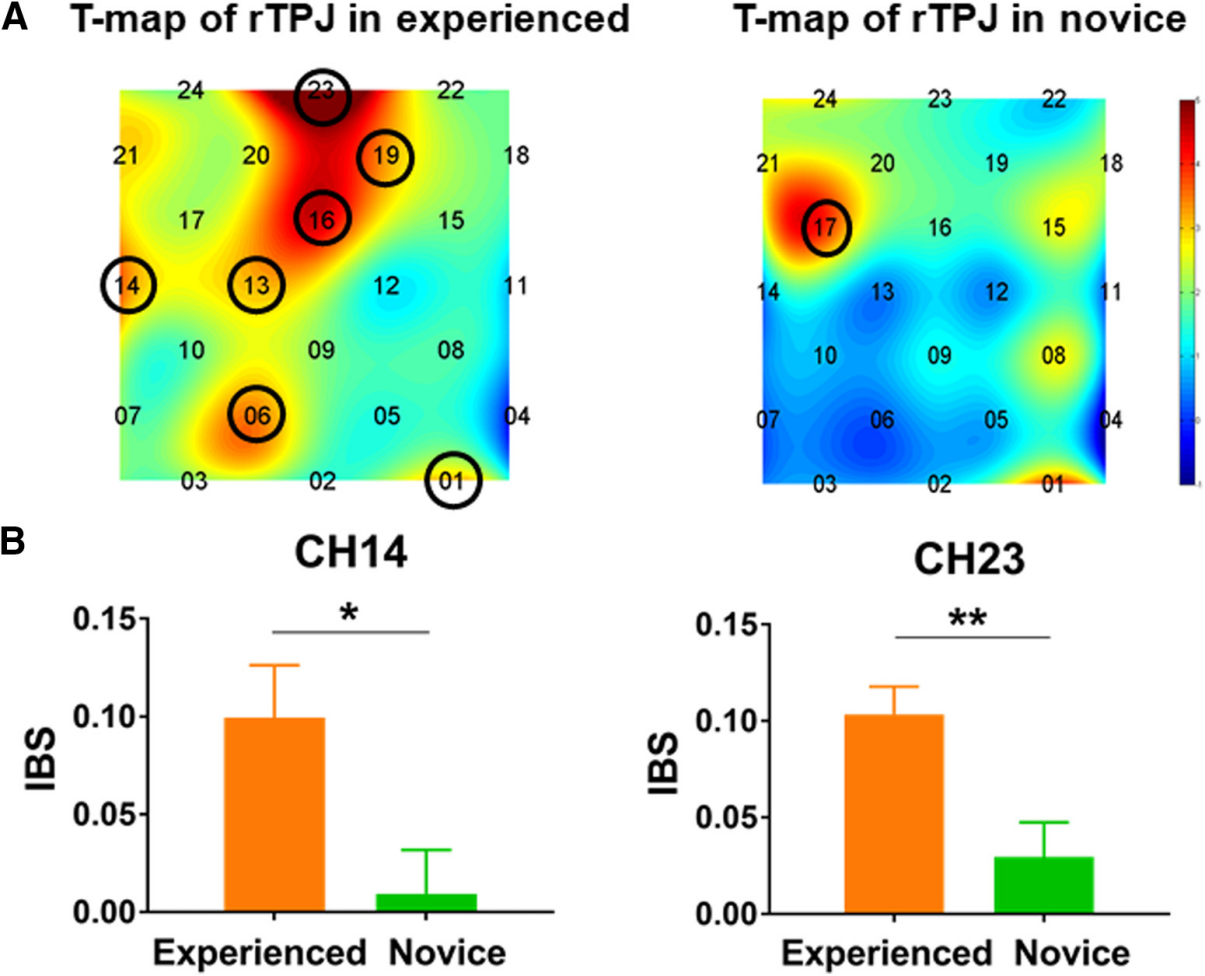
Comparison of the IBS achieved in the two groups. ***A***, CH1, CH6, CH13, CH14, CH16, CH19, and CH23 in the experienced counselor group and CH17 in the novice counselor group showed significant synchronization (FDR corrected). ***B***, IBS was significantly larger on CH14 and CH23 in the experienced counselor group compared with that in the novice counselor group (period = 22.24–41.98 s); *p <* *0.05, **p <* *0.01. Error bars indicate SEs.

To identify the frequency ranges specifically associated with psychological counseling, a series of *t* tests were conducted on the task-related IBS for the full-time range (10–100 s, 0.01–0.1 Hz). Significant differences between 24.96 and 33.32 s were observed. The frequencies around these two values were subsequently investigated, and those with *p* values <0.05 were selected, resulting in frequencies ranging from 22.24 to 41.98 s. A one sample *t* test was then conducted on task-related IBS values within the frequency range. A significantly larger task-related IBS was found on CH1, CH6, CH13, CH14, CH16, CH17, CH19, CH20, CH21, and CH23 in the experienced counselor group, and on CH1, CH8, CH15, CH17, CH18, CH20, CH21, and CH24 in the novice counselor group. The resulting *p* values were corrected using the FDR method across all CHs and all frequencies (*p *<* *0.05). The number of *p* value is 48 (CHs). All of these *p* values were FDR corrected at one time. After this FDR correction, a significant increase in IBS was confirmed on CH1, CH6, CH13, CH14, CH16, CH19, and CH23 in the experienced counselor group, and on CH17 in the novice counselor group (Fig. 3*A*).

A series of *t* tests on IBS values were conducted to determine the difference between the two groups. IBS in the rTPJ in the experienced counselor group was significantly larger than that in the novice counselor group on CH14 and CH23 (Fig. 3*B*). No significant differences were observed for other CHs (CH14, *t*_(28)_ = 2.59, *p *=* *0.015; CH23: *t*_(28)_* *=* *3.16, *p *=* *0.004).

**Figure 4.**
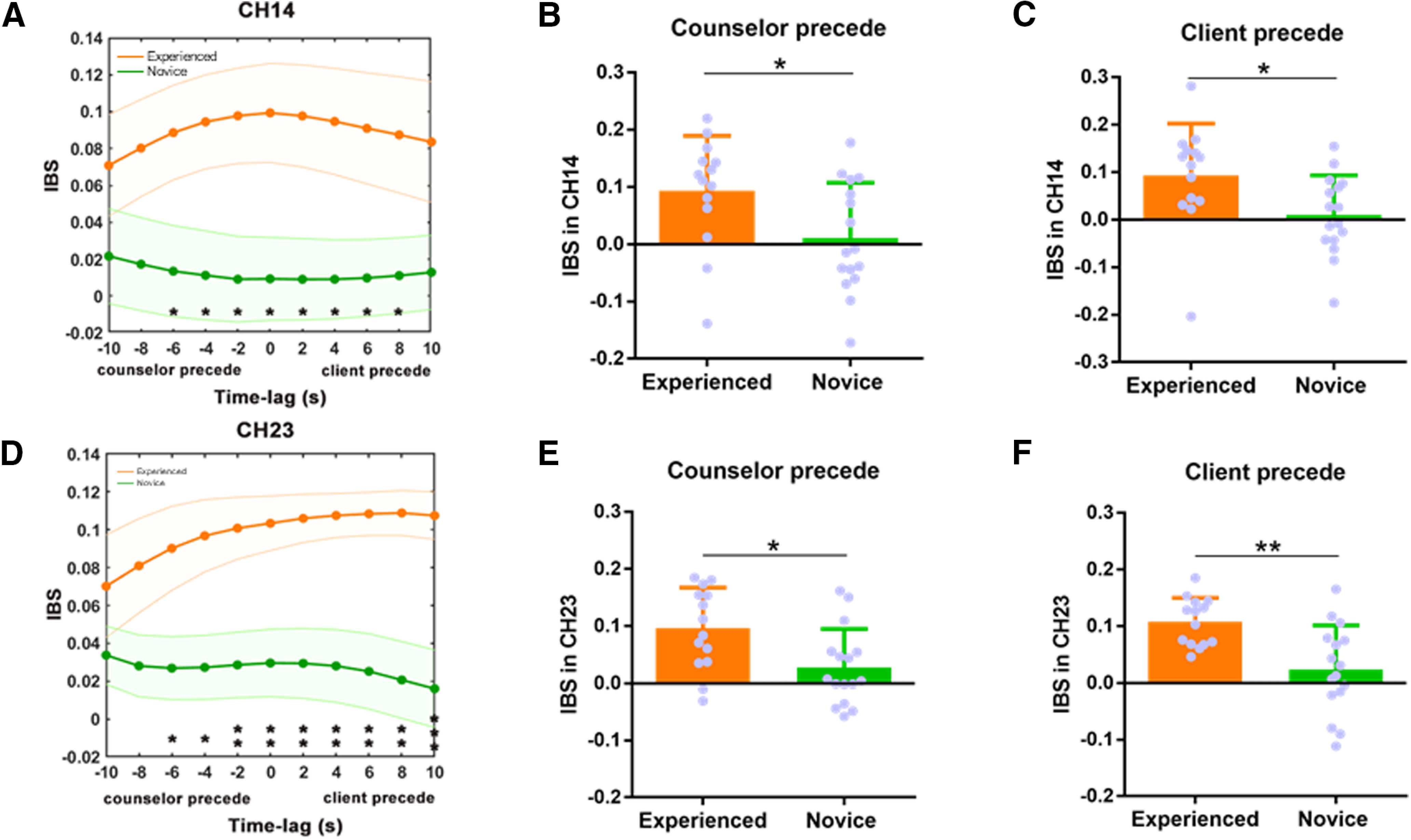
Comparison of time-lagged IBS between the two groups. ***A–C***, The difference in task-related IBS between the two groups when the client’s brain activity preceded that of the counselor by −10−10 s (with 2-s intervals) on CH14. ***D–F***, The differences in task-related IBS within the two groups when a client’s brain activity preceded that of the counselor by −10−10 s (with 2-s intervals) on CH23. *p <* *0.05, **p <* *0.01.

The time-lag results revealed that task-related IBS in the experienced counselor group was significantly larger than that in the novice counselor group when the client’s brain activity preceded that of the counselor by 2, 4, 6, and 8 s on CH14; and 2, 4, 6, 8, and 10 s on CH23 (CH14: *t*_2s(28)_ =* *2.53, *p *=* *0.017; *t*_4s(28)_* *=* *2.42, *p *=* *0.022; *t*_6s(28)_* *= 2.26, *p *=* *0.032; *t*_8s(28)_* *=* *2.08, *p *=* *0.047; *t*_10s(28)_* *=* *1.89, *p *>* *0.05; CH23: *t*_2s(28)_* *=* *3.32, *p *=* *0.003; *t*_4s(28)_* *=* *3.41, *p *=* *0.002; *t*_6s(28)_* *=* *3.49, *p *=* *0.002; *t*_8s(28)_* *=* *3.61, *p *=* *0.001; *t*_10s(28)_* *=* *3.67, *p *=* *0.001). Task-related IBS was subsequently averaged among the values when the client’s brain activity preceded that of the counselor by 2, 4, 6, 8, and or 10 s. A comparison of the mean IBS values between the two groups showed that IBS was significantly larger in the experienced counselor group relative to the novice counselor group (CH14: *t*_(28)_ =* *2.35, *p *=* *0.026; CH23: *t*_(28)_ = 3.60, *p *=* *0.001; Fig. 4*A*,*C*,*D*,*F*).

Task-related IBS in the experienced counselor group was significantly larger than that in the novice counselor group when the counselor’s brain activity preceded that of the clients by 2, 4, and 6 s on CH14 and CH23 (CH14: *t*_2s(28)_* *=* *2.56, *p *=* *0.016; *t*_4s(28)_* *=* *2.37, *p *=* *0.025; *t*_6s(28)_* *= 2.10, *p *=* *0.044; *t*_8s(28)_* *=* *1.72, *p *>* *0.05; *t*_10s(28)_* *=* *1.30, *p *> 0.05; CH23: *t*_2s(28)_* *=* *3.00, *p *=* *0.006; *t*_4s(28)_* *=* *2.73, *p *= 0.011; *t*_6s(28)_* *=* *2.32, *p *=* *0.028; *t*_8s(28)_* *=* *1.82, *p *>* *0.05; *t*_10s(28)_* *= 1.20, *p *>* *0.05). The task-related IBS among the values for which the counselor’s brain activity preceded that of the clients by 2, 4, and 6 s were averaged, and the average IBS was compared between groups. A significantly larger IBS was observed in the experienced counselor group relative to that in the novice counselor group (CH14: *t*_(28)_ = 2.35, *p *=* *0.026; CH23: *t*_(28)_ = 2.70, *p *=* *0.012; Fig. 4*A*,*B*,*D*,*E*).

### Neural-behavioral correlation

**Figure 5.**
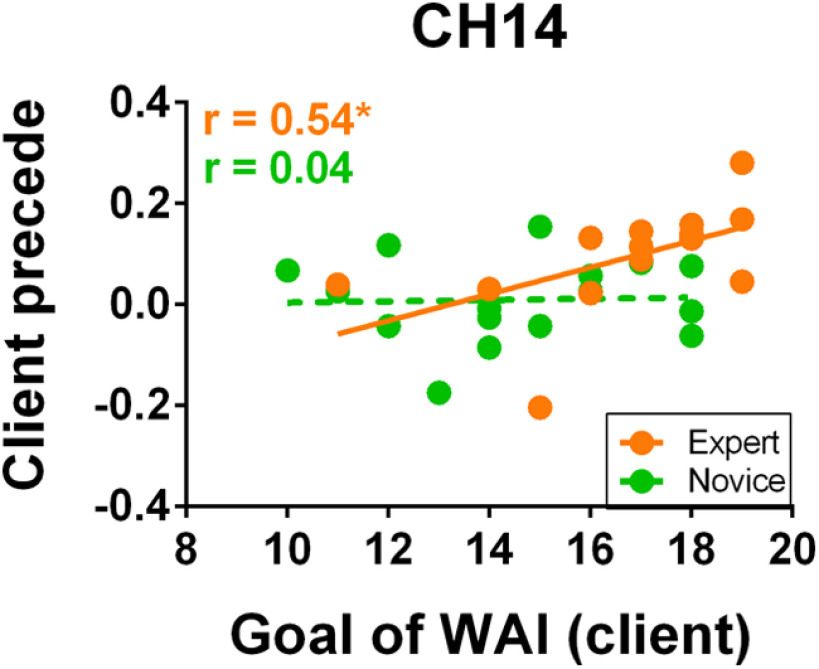
IBS-behavioral correlation. The correlation between the averaged time-lagged IBS of the rTPJ and the goal component of the WAI-SR in the experienced-counselor group. A positive correlation was found in the experienced counselor group; *p <* *0.05.

We performed Pearson correlational analyses among WAI total scores (and three subscales), IBS and time-lagged IBS (counselor proceeded and client proceeded) on CH14 and CH23 in two groups respectively. In the experienced counselor group, the result showed that when the client’s brain activity preceded that of the counselor for the averaged time (2, 4, 6, and 8 s), a significant correlation was observed on CH14 between IBS and goal development, a dimension of the WAI-SR (*r *=* *0.54, *p *=* *0.032, uncorrected; Fig. 5). However, this *p* value (0.032) had not passed the FDR correction, which might partly because of the limited sample size (*n* = 14, in the experienced counselor group). This result might indicate that IBS in the experienced-counselor group was related to the goal developed between the counselor and client. However, no significant correlations between IBS, time-lagged IBS and WA were found on CH23 in the experienced counselor group. Moreover, in the novice counselor group, there was no association between IBS, time-lagged IBS and WA on both CH14 and CH23.

## Discussion

This study aimed to investigate the role of the counselor’s experience in the formation of WA with clients in the first session of psychological counseling. First, psychological counseling elicited synchronous brain activity from the rTPJ across the counselor-client dyads. Second, significant increases in the WA and IBS of the rTPJ were observed in the experienced counselor group relative to that of the novice counselor group. Third, the detected time-lagged IBS in the experienced counselor group was significantly correlated with the goal component of the WA that was developed between the counselor and client in the first session.

A recent debate about the influence of a counselor’s experience on WA focused on whether experienced counselors are better than inexperienced ones at building relationships or promoting clinical improvement. Our behavioral results contribute to this debate by demonstrating that the experienced-counselor group established a significantly greater WA than did the novice-counselor group, at least in the first session. Meanwhile, there was no significant difference in clinical improvement based on the SOS-10 between the two groups after the first session. This inconsistent results with previous studies might partly because of the different therapeutic orientation and WAI rating points (Hersoug et al., 2001; a sample of 59 primarily psychodynamic therapists with WAI rating in session three and session 12; Gelso et al.,2005; a sample of 80 therapists with highly diverse orientation and therapists rated WAI with their previous works). Our sample included eight counselors (three experienced counselors and five novice counselors) with integrative orientation. They provided brief psychological service for college students and were trained to focus on the emotional states and reported troubles of the client, as well as the client’s expectation for pursuing counseling. In our opinion, therapists’ integrative orientation and program training might contribute to their empathizing on goal forming, emotional feedback during clinical work, which initiated the increased WA scores in the experienced counselor group, at least in the first session.

Moreover, our study found significant increases in goal and task (two dimensions of WAI) measures in the experienced counselor group compared with those in the novice counselor group during the first session. This result corroborates previous findings demonstrating that clinical experience contributes to goal and task aspects during the initial stage of psychological counseling (Mallinckrodt and Nelson, 1991; Oddli and Halvorsen, 2014). Therefore, in comparison to novice counselors, experienced counselors may be able to initiate a better start with their clients via more effective goal forming and task assignments.

In our study, we observed that psychological counseling elicited IBS of the rTPJ across the counselor-client dyads and that this IBS was significantly increased in the experienced counselor group relative to that in the novice group. Previous studies have shown that IBS is related to the level of understanding and emotional interaction (Mu et al., 2018), successful communication (Stephens et al., 2010), and interpersonal closeness/connectedness (Kinreich et al., 2017) between communicators. Accordingly, we contributed the increased IBS to the tighter interpersonal closeness/connectedness or better alliance/emotional interaction which was triggered by the experienced counselor versus the novice counselor. In fact, this result was consistent with previous studies on IBS in psychological process. For example, Zhang et al. (2018) found the increased IBS in psychological counseling process versus chatting process, which was associated with WA (Zhang et al., 2018). Taken together, IBS might be influenced by dyads’ closeness/connectedness or better alliance during the psychological counseling process. These findings might provide neural evidence to suggest that an experienced counselor may facilitate communication or an alliance with clients, even in the first session of psychological counseling.

Indeed, IBS was observed in the rTPJ, a brain region linked to the regulation of behaviors such as building positive relationships (Kinreich et al., 2017), cognitive empathy (Atique et al., 2011), and shared intentionality (Koster-Hale et al., 2013; Dai et al., 2018). Our observation of greater IBS of the rTPJ in the experienced-counselor group supports the notion that a counselor’s level of experience is important in establishing connectedness or positive alliance during the first session of psychological counseling.

Moreover, even in the novice counselor group, we found increased IBS comparing with baseline. This increased IBS might partly because of the face-to-face communication as well as the (novice level) use of counseling skills. On account of lacking non-counselor group (without any theoretical training of psychological counseling before the study), we could not separate the influence of individual characteristics (personality, social skills; present before any training) and professional knowledge (e.g., counseling skills present on a novice level). However, this result put hint on the possible influence of the novice level counseling skills on brain synchronization between the psychological counseling dyads.

Another notable observation made in the experienced counselor group was that IBS was correlated with the goal component of WA when the client’s brain activity preceded that of the counselor. This finding illustrates the possibility that brain synchronization within a dyad may induce concomitant development of the WA. Indeed, previous studies have found that in initial psychological counseling sessions, experienced counselors and their clients spend relatively little time on the explicit discussion of goals; however, external observation analyses have indicated that psychotherapists and their clients do clearly work toward goal establishment (Oddli et al., 2014). Our study provides further neural evidence to support this valuable implicit process (Oddli and Halvorsen, 2014). As such, even in the first session of psychological counseling, brain-to-brain coupling between experienced counselors and their clients may facilitate goal formation. In other words, “the more tightly the client and counselor’s brains are coupled, the better the alliance.” (Koole and Tschacher, 2016).

More specifically, the goal component of WA was only associated with time-lagged counselor-client brain synchronization when the client’s brain activity preceded that of the counselor. The direction of time-lagged IBS implied that the primary flow of information occurred from client to counselor. This result is consistent with a recent debate about the role of a counselor’s expertise, which emphasized that expert/experienced counselors must be able to adapt to different types of clients, as well as being responsive and collaborative (Hill et al., 2017b). In details, experienced psychotherapists reported that they used moment-to-moment cues (e.g., emotional expression, body postures) and tried to be attentive to their clients’ reactions, approvals or rejections, although they were not openly discussed (Oddli and Halvorsen, 2014). Our study supported the potential adaptive mechanism embedded in psychological counseling between counselor-client dyads.

Our study has several main limitations. First, the WA and IBS were only compared between experienced versus novice counselors with their respective clients in the first session of psychological counseling. Further studies should thus focus on WA development across the entire counseling process, especially among the non-counselors, novice-counselors, experienced counselors, and expert counselors with their clients; this may further elucidate the role of expertise in building effective WAs with clients. Second, the fNIRS we used in this study was only able to detect changes in blood flow concentration at the cortical level; this limited the breadth of exploration for the neural events associated with relationship development between clients and their counselors during the counseling process. Accordingly, we limited our focus to the rTPJ. Future studies should, therefore, monitor additional brain areas to better characterize how neural engagement between the client and psychotherapist differs according to the counselor's level of experience. Third, our study had only 30 counselor-client dyads, which might underpower the evidence about the role of experience in the alliance formation. Fourthly, our study examined only the first counseling session, which limited the exploration of whether IBS during counseling could contribute to therapeutic benefit or not. Future studies should consider the relationship between IBS and clinical changes across multiple sessions. Lastly, in our study, participants consist of 8 counselors and 30 clients, which contributed to 16 dyads in the novice counselor group and 14 dyads in the experienced counselor group. Although we found no significant difference in IBS and WA across different counselors in two groups separately, the linear mixed models (LMMs) might be the better analysis method in future study.

## Conclusion

To the best of our knowledge, this is the first study to have used fNIRS hyperscanning for correlating the extent of client-counselor WA formation and brain interaction with the counselor’s level of experience during the first session of psychological counseling. Importantly, our findings revealed that IBS of the rTPJ, which might associate with the *goal* component of the WA, was significantly greater in the experienced counselor group than in the novice counselor group. This result provided pieces of neural evidence that the experienced counselors could build a better relationship or alliance with the clients than novice counselors at least in first session. Meanwhile, a potential adaptive mechanism is embedded in psychological counseling.

### Data transparency statement

The data from our study reported in this article have not been previously published. The models and relationships examined in the present article have not been examined in any other articles that were submitted for review. The authors were unaware of any publications examining similar topics using the datasets included in the study of the present article at the time of this submission.

## Synthesis

Reviewing Editor: Macià Buades-Rotger, University of Luebeck Decisions are customarily a result of the Reviewing Editor and the peer reviewers coming together and discussing their recommendations until a consensus is reached. When revisions are invited, a fact-based synthesis statement explaining their decision and outlining what is needed to prepare a revision will be listed below. The following reviewer(s) agreed to reveal their identity: NONE.

Dear authors,

Thank you for your clear and interesting manuscript on interpersonal neural synchrony in novice and expert counselor-client dyads during their initial counseling session. While the results from experienced dyads are a replication of previous studies, observations on novice counselors are a new finding that constitute an informative addition to the field. I will hence recommend the manuscript for publication once you have addressed the following issues:

Major points:

- The significance statement should discuss the neural mechanisms is slightly greater depth, as befits a neuroscience journal. Also, the sentence “It also suggests that a potential adaptive mechanism is embedded in psychological counseling” is vague and not justified by the data. Please specify what is meant by this and explicitly state how it is supported by the results.

- It was surprising that there was no INS within novice dyads (Figures 3-4). As the authors discuss, INS has been observed in simple conversations. In a previous publication that used very similar protocols and questionnaires, this was observed as well for the chatting condition. Figures 3 and 4 suggest there was little to no synchrony in the novice group. The authors should address the potential reasons underlying this finding in this group in the discussion section, citing additional literature and/or making testable predictions for future studies if pertinent.

- Did the novice counselors have any theoretical training prior to the study or their counseling work? If not, they seem much more similar to non-counselors. Possibly, in individuals who had theoretical training but start their working experience, INS would be higher due to the (novice level) use of counseling skills. This might help explaining the conflicting literature on the effects of clinician work experience by separating individual characteristics (personality, social skills; present before any training) and professional knowledge (counseling skills; present on a novice level after theoretical training). To make this clear, especially as an addition to the mentioned discussion, the authors should consider stating this explicitly.

- The analysis suggests independence of individual data sets. However, each counselor was examined 3-5 times. This needs to be accounted for in the analysis and addressed in the discussion.

- The interpretations are limited by the design that only examined one counseling session. Since change in well-being occurs slower across multiple sessions, it remains unknown whether INS during counseling is important for therapeutic benefit or not. The authors should explicitly address this issue in the limitations.

- The number of tests and corrections is unclear. For example, in section 2.5.3, it seems that 2*10*6 values were correlated (2 channels, 10 time shift steps, WAI total score + 3 subscales + 2 SOS timepoints). If this is the case then it can be assumed there was some form of correction used for the large number of tests? Please state explicitly in the methods and in line 263: “After correction for multiple comparisons (p < 0.05).”

- Please provide exact p-values up to three decimal places (p=.XXX) rather than approximate ones (p<.XX).

- Two abbreviations are used for brain synchronization between subjects. INS is used throughout the main text; IBS is used in the figures (and once each in the methods and results section). Please keep this consistent throughout the text.

- Line 77: Please rewrite and make the transition clearer. We suggest summing up the main points described previously, leading to the study idea.

- Line 96: The authors do not discuss a possible bias due to all clients being female. Do they believe this could have an influence on the results?

- Line 108: The authors do not report the gender of the experienced counselors. Please add.

- Line 109: How is good reputation of the experienced counselors defined? Please clarify or delete.

- Line 109: Hours of experience vary greatly between experienced counselors. Can the authors still justify the homogeneity in this group?

- Line 206: Please rephrase the following sentence, as it is unclear what procedure the authors exactly followed: “Second, the frequencies that were around these two frequencies and meanwhile whose p values were < 0.05 were selected, resulting in the frequency range (0.02-0.05Hz).”

- Line 223: What do the authors mean with >under different conditions<?

- Line 256: Which time-range is meant here?

- Line 316: How can the authors unambiguously define their findings to be emergent from rTPJ?

Minor points

- Add spaces between >spectroscopy(fNIRS)< (e.g., line 6)

- Line 22: >in the first session<

- Line 204: Write out this abbreviation at its first use (>CH<).

## Author Response

Response to Editor and Reviewer

We wish to thank the editor and the reviewers for their insightful comments and constructive suggestions concerning our manuscript titled “Experience-dependent counselor-client brain synchronization during psychological counseling” (eN-NWR-236-20). The manuscript has been revised accordingly (the novel/changed parts are highlighted in blue). In what follows, we respond to all the editor’s and reviewers’ points.

Editors:

Thank you for your clear and interesting manuscript on interpersonal neural synchrony in novice and expert counselor-client dyads during their initial counseling session. While the results from experienced dyads are a replication of previous studies, observations on novice counselors are a new finding that constitute an informative addition to the field. I will hence recommend the manuscript for publication once you have addressed the following issues.

Response: We have revised our manuscript following the new comments and suggestions. We hope our revisions will satisfy the editors and the reviewer. Particularly, we (1) updated our significance statement, introduction and study limitation (2) clarified more information about the data analysis and participant information, and (3) added more NIRS technical details. Detailed responses are as follows.

1- The significance statement should discuss the neural mechanisms is slightly greater depth, as befits a neuroscience journal. Also, the sentence “It also suggests that a potential adaptive mechanism is embedded in psychological counseling” is vague and not justified by the data. Please specify what is meant by this and explicitly state how it is supported by the results.

Response: thanks for your careful suggestion. We now updated the significance statement in revised manuscript as following (see p.1, line 20-31):

"Recent expanding nascent studies on interpersonal brain synchronization (IBS) during the interpersonal communication process using functional near-infrared spectroscopy(fNIRS) have hinted at the possible neural substrates underlying the effective relationship/alliance between the counselor-client dyads. By using fNIRs, our study found that the experienced counselors could build better alliance and stronger brain synchronization with the clients vs. novice counselors, at least in the first session. Moreover, IBS was observed in the right temporo-parietal junction (rTPJ), a brain region linked to the regulation of behaviors such as building positive relationships or cognitive empathy. This result supports the notion that a counselor’s level of experience is important in establishing connectedness or positive alliance during the first session of psychological counseling.”

2- It was surprising that there was no INS within novice dyads (Figures 3-4). As the authors discuss, INS has been observed in simple conversations. In a previous publication that used very similar protocols and questionnaires, this was observed as well for the chatting condition. Figures 3 and 4 suggest there was little to no synchrony in the novice group. The authors should address the potential reasons underlying this finding in this group in the discussion section, citing additional literature and/or making testable predictions for future studies if pertinent.

Response: Thanks for your careful suggestion. In fact, a significantly larger task-related IBS was found on channel (CH)1, CH6, CH13, CH14, CH16, CH17, CH19, CH20, CH21, and CH23 in the experienced counselor group, and on CH1, CH8, CH15, CH17, CH18, CH20, CH21, and CH24 in the novice counselor group. After the FDR correction, a significant increase in IBS was confirmed on CH1, CH6, CH13, CH14, CH16, CH19, and CH23 in the experienced counselor group, and on CH17 in the novice counselor group (Fig. 3A). Figure 3 and 4 suggest little synchrony in the novice group, however, a significant increase in IBS comparing with baseline was confirmed on CH17 in this group. Previous studies found increased IBS in face-to-face communication (Jiang et al., 2012; Nowaza et al., 2016) or face-to-face psychological counseling (Zhang et al., 2018). In our study, we compared only the novice counselor group and the experienced counselor group, which limited our explanations of the difference among simple conversation (chatting), novice-counselor counseling and experienced-counselor counseling. In future study, we might compare the groups of the non-counselors, the novice-counselors, the experienced counselors and the expert counselors with their clients; this may further elucidate the role of expertise in building effective WAs with clients. In the revised manuscript, we had updated this part in discussion as following (see p.18, line 424-430):

"First, the WA and IBS were only compared between experienced versus novice counselors with their respective clients in the first session of psychological counseling. Further studies should thus focus on WA development across the entire counseling process, especially among the non-counselors, novice-counselors, experienced counselors and expert counselors with their clients; this may further elucidate the role of expertise in building effective WAs with clients.”

3- Did the novice counselors have any theoretical training prior to the study or their counseling work? If not, they seem much more similar to non-counselors. Possibly, in individuals who had theoretical training but start their working experience, INS would be higher due to the (novice level) use of counseling skills. This might help explaining the conflicting literature on the effects of clinician work experience by separating individual characteristics (personality, social skills; present before any training) and professional knowledge (counseling skills; present on a novice level after theoretical training). To make this clear, especially as an addition to the mentioned discussion, the authors should consider stating this explicitly.

Response: Thanks for your careful and insightful suggestions. The novice counselors in our study were first-year graduate students with some clinician work experience (15-23 hours) under the supervision. They were trained by professional counseling knowledges (e.g., counseling skills, therapeutic theories, counseling ethics, interviewing methods and so on). They are not non-counselors. In our study, we found a significant increase in IBS on CH17 in the novice group after the FDR correction. This might due to the (novice level) use of counseling skills or just face-to-face communications. In fact, we could not separate the influence of counselors’ individual characteristics from their counseling skills in the present study, since only face-to-face communication might trigger increased IBS (e.g., Jiang et al., 2012; Nowaza et al., 2016), which might be affected by individual characteristics of dyads. In order to explain the conflicting literature on the effects of clinician work experience by separating individual characteristics (personality, social skills; present before any training) and professional knowledge, in future study, we should investigate the WA and IBS among at least three groups of the non-counselors, the novice-counselors and the experienced counselors. Accordingly, we supplemented this part in the discussion as following (see p. 17, line 398-406):

"Moreover, even in the novice counselor group, we found increased IBS comparing with baseline. This increased IBS might partly due to the face-to-face communication as well as the (novice level) use of counseling skills. On account of lacking non-counselor group (without any theoretical training of psychological counseling prior to the study), we could not separate the influence of individual characteristics (personality, social skills; present before any training) and professional knowledge (e.g., counseling skills present on a novice level). However, this result put hint on the possible influence of the novice level counseling skills on brain synchronization between the psychological counseling dyads.”

4- The analysis suggests independence of individual data sets. However, each counselor was examined 3-5 times. This needs to be accounted for in the analysis and addressed in the discussion.

Response: Thanks for your careful suggestion. In our study, participants consist of 8 counselors and 30 clients, which contributed to 16 dyads in the novice counselor group and 14 dyads in the experienced counselor group. In fact, the analysis was not independent of individual data sets. In the revised manuscript, we had updated the description of our analysis (see the data analysis part). Additionally, in order to test the difference between counselors, we performed the one-way ANOVA with Counselor (counselor 1 vs. counselor 2 vs. counselor 3) on the WA and IBS in experienced counselors’ group and found no significant effect involving Counselors (all ps > 0.18); similarly, we performed the one-way ANOVA with Counselor (counselor 1 vs. counselor 2 vs. counselor 3 vs. counselor 4 vs. counselor 5) on the WA and IBS in novice counselors’ group and found no significant effect involving Counselors (all ps > 0.21). However, to directly examine differences between conditions in brain synchronization, we might better apply the linear mixed models (LMMs) in the respective CHs as the dependent variable. Accordingly, we added the limitation in our discussion part as following (see p.19 line 450-455):

"Lastly, in our study, participants consist of 8 counselors and 30 clients, which contributed to 16 dyads in the novice counselor group and 14 dyads in the experienced counselor group. Though we found no significant difference in IBS and WA across different counselors in two groups separately, the linear mixed models (LMMs) might be the better analysis method in future study.”

5- The interpretations are limited by the design that only examined one counseling session. Since change in well-being occurs slower across multiple sessions, it remains unknown whether INS during counseling is important for therapeutic benefit or not. The authors should explicitly address this issue in the limitations.

Response: Thanks for your helpful suggestion. In the revised manuscript, we have supplemented this limitation (see p. 19, line 447-450).

"Fourthly, our study examined only the first counseling session, which limited the exploration of whether IBS during counseling could contribute to therapeutic benefit or not. Future studies should consider the relationship between IBS and clinical changes across multiple sessions.”

6- The number of tests and corrections is unclear. For example, in section 2.5.3, it seems that 2*10*6 values were correlated (2 channels, 10 time shift steps, WAI total score + 3 subscales + 2 SOS timepoints). If this is the case then it can be assumed there was some form of correction used for the large number of tests? Please state explicitly in the methods and in line 263: “After correction for multiple comparisons (p < 0.05).”

Response: Thanks for your careful suggestion. First，during the analysis of IBS, a one sample t-test was then conducted on task-related IBS values within the frequency range. A significantly larger task-related IBS was found on channel CH1, CH6, CH13, CH14, CH16, CH17, CH19, CH20, CH21, and CH23 in the experienced counselor group, and on CH1, CH8, CH15, CH17, CH18, CH20, CH21, and CH24 in the novice counselor group. The resulting p values were corrected using the false discovery rate (FDR) method across all CHs (p < 0.05). The number of p value is 48 (CHs). All of these p values were FDR corrected at one time.

Second, in section 2.5.3, we chose only the CHs with (1) significant increased IBS comparing with the baseline and (2) significant IBS differences in two groups. Afterward, we performed Pearson correlational analyses among WAI total scores, IBS and time-lagged IBS (including client preceded and counselor preceded time-lagged IBS) on CH14 and CH23 in two groups respectively. For each CHs of each group, 21 values [3 (IBS and time-lagged IBS) * 4 (WAI total score and 3 subscales) were correlated. In the experienced counselor group, the result showed that when the client’s brain activity preceded that of the counselor for the averaged time (2, 4, 6, and 8 s), a significant correlation was observed on CH14 between IBS and goal development, a dimension of the WAI-SR (r = 0.54, p = 0.032, uncorrected, Fig. 5). Accordingly, the resulting p values were corrected using the false discovery rate (FDR) method. However, this p value (0.032) had not passed the FDR correction. This might partly due to the limited sample size (n= 14, in the experienced counselor group).

Accordingly, we updated the details above in revised manuscript (see p.13 line 289-292; p.12 line 260-263; p.14-15, line 330-343):

"The resulting p values were corrected using the false discovery rate (FDR) method across all CHs (p < 0.05). The number of p value is 48 (CHs). All of these p values were FDR corrected at one time.”

"We chose only the CHs with (1) significant increased IBS comparing with the baseline and (2) significant IBS differences in two groups. Pearson correlational analyses were performed to explore the relationships among IBS, time-lagged IBS, and WA...We performed Pearson correlational analyses among WAI total scores, IBS and time-lagged IBS on CH14 and CH23 in two groups respectively. In the experienced counselor group, the result showed that when the client’s brain activity preceded that of the counselor for the averaged time (2, 4, 6, and 8 s), a significant correlation was observed on CH14 between IBS and goal development, a dimension of the WAI-SR (r = 0.54, p = 0.032, uncorrected, Fig. 5). However, this p value (0.032) had not passed the FDR correction, which might partly due to the limited sample size (n = 14, in the experienced counselor group).”

7- Please provide exact p-values up to three decimal places (p=.XXX) rather than approximate ones (p<.XX).

Response: Now we have revised all p-values to exact three decimal places (see revised manuscript).

8- Two abbreviations are used for brain synchronization between subjects. INS is used throughout the main text; IBS is used in the figures (and once each in the methods and results section). Please keep this consistent throughout the text.

Response: Thanks for your careful suggestion. We have changed all the abbreviations into IBS and kept these two abbreviations consistent in our revised manuscript (see IBS in the manuscript and figures).

9- Line 77: Please rewrite and make the transition clearer. We suggest summing up the main points described previously, leading to the study idea.

Response: We have updated the transition part in revised manuscript (see p. 5, line 86-95):

"In short, previous researches kept controversial about whether experienced counselors can build a better relationship or alliance with clients than novice counselors can. IBS during the interpersonal communication process provided possible neural indicators of effective alliance/relationship between counselor-client dyads. Accordingly, we aimed to compare IBS as well as the relationship qualities between the experienced counselor-client dyads and the novice counselor-client dyads by using fNIRS hyperscanning, which is a safe, non-invasive imaging modality (Ferrari and Quaresima, 2012) that uses NIR light projected to (source) and from (detractor) tissues to quantitatively monitor the levels of cortical oxyhemoglobin (oxyHb) and deoxyhemoglobin (deoxyHb).”

10- Line 96: The authors do not discuss a possible bias due to all clients being female. Do they believe this could have an influence on the results?

Response: Thanks for your insightful suggestion. Previous studies found that synchronous brain activity during cooperative interpersonal activities depends on gender of partner (e.g., Chen Li & Hu, 2015; Lu, Deng, & Hao, 2020), we chose only female participants in our study (female client- female counselor dyads) to avoid the gender effect on brain synchronization. Based on previous studies, we speculated that the IBS in same-gender counselor-client dyads might increase comparing with different-gender dyads. However, when comparing the IBS between the novice-counselor and the experienced-counselor groups with same gender, this gender bias can be eliminated.

Accordingly, we added the description about choosing only female clients and female counselors as following (see p.6 line 114-118):

"Previous studies found that synchronous brain activity during cooperative interpersonal activities depends on gender of partner (e.g., Chen Li & Hu, 2015; Lu, Deng, & Hao, 2020). In our study, we chose only female participants (female clients and female counselors in two groups) to avoid the gender effect on brain synchronization.”

11- Line 108: The authors do not report the gender of the experienced counselors. Please add.

Response: We have added the description about the gender of the experienced counselors (see p. 6, line 125).

12- Line 109: How is good reputation of the experienced counselors defined? Please clarify or delete.

Response: Thanks for your careful suggestion. we now have deleted this description (see p. 5, line 125).

"For the experienced counselor group, we recruited three female licensed psychological counselors (age range: 29-45 years; Mage: 34.7 years) and 600-4000 hours’ experience in providing psychological counseling.”

13- Line 109: Hours of experience vary greatly between experienced counselors. Can the authors still justify the homogeneity in this group?

Response: Thanks for your careful suggestion. In China, licensed counselors were required to have at least 250 hours clinical practice according to the Registered psychologist system of Chinese Psychological Society. The experienced counselors in our study were licensed psychological counselors who had been trained by a college counseling service program. However, their clinical practice hours varied from 600 to 4000 hours. To exclude the possibility that the IBS might differ across counselors, we performed one-way ANOVA with Counselor (counselor 1 vs. counselor 2 vs. counselor 3) on the WA and IBS in experienced counselors’ group and found no significant effect involving Counselors. In future studies, we should balance the clinical practice hours of the counselors. We added this description in our result part (see p. 11, line 235-239):

"To exclude the possibility that the IBS might differ across counselors, we performed one-way ANOVAS with Counselor (counselor 1 vs. counselor 2 vs. counselor 3) on the IBS and WA in experienced counselors’ group and found no significant effect involving Counselors (all ps > 0.18).”

14- Line 206: Please rephrase the following sentence, as it is unclear what procedure the authors exactly followed: “Second, the frequencies that were around these two frequencies and meanwhile whose p values were < 0.05 were selected, resulting in the frequency range (0.02-0.05Hz).”

Response: We revised this sentence as following (see p. 10, line 227-229) :

"Second, we selected the frequencies that were around 0.04Hz and 0.03 Hz as well as their p values were < 0.05. Then we obtained the frequency range from 0.02 to 0.05 Hz.”

15- Line 223: What do the authors mean with >under different conditionsResponse: we revised this phase into “under the ten conditions” (see p. 11, line 247).

16- Line 256: Which time-range is meant here?

Response: Full time-range varies from 10s to 100s (0.01-0.1 Hz). Data below 0.01 Hz were not considered to remove very low-frequency fluctuations. Data above 0.1 Hz were also considered to exclude noises such as cardiac activity (∼ 0.8-2.5 Hz) and respiratory activity (∼ 0.15-0.3 Hz) (Guijt et al. 2007; Tong et al. 2011; Barrett et al. 2015). We have described this part in data analysis part as following: “First, to identify the frequency ranges that were specifically associated with counseling, the data from the two groups were combined, and the IBS during the baseline stage was subtracted to obtain the task-related IBS. We then conducted one sample t-test on the time-averaged task-related IBS from both groups, along with the full frequency range (0.01-0.1 Hz) (according to previous fNIRS-based hyperscanning studies, e.g., Jiang et al., 2012). A threshold of p < 0.0005 was applied to the results according to the method reported by Zheng et al. (2018). No further correction for multiple comparisons was applied because this analysis was only used to identify the pattern along the frequency range, rather than to obtain final results. Only two frequencies (i.e., 0.04Hz and 0.03Hz) had channel (CH) combinations whose p value survived the thresholding.” Accordingly, in the result part, we updated the description as following (p.13, line 282):

"To identify the frequency ranges specifically associated with psychological counseling, a series of t-tests were conducted on the task-related IBS for the full-time range (10-100 s, 0.01-0.1Hz). Significant differences between 24.96 and 33.32 s were observed.”

17- Line 316: How can the authors unambiguously define their findings to be emergent from rTPJ?

Response: Based on previous studies about the role of IBS in psychological counseling (Zhang et al., 2018), the rTPJ (Fig. 1C) was selected as the region of interest and a 4×4 probe set (eight emitters and eight detectors, forming 24 measurement channels [CHs]) was placed over the rTPJ regions referenced to P6 (10/20 international system) (Fig. 1B). Additionally, to determine the correspondence between the NIRS CHs and the measurement points on the cerebral cortex, the virtual registration method was used to make sure about the position of rTPJ. We have added this supplemental description in our revised manuscript (see p. line 8, 181-183):

"To determine the correspondence between the NIRS CHs and the measurement points on the cerebral cortex, the virtual registration method was used (Singh et al., 2005; Tsuzuki et al., 2007).”

18-Minor points

- Add spaces between >spectroscopy(fNIRS)< (e.g., line 6)

- Line 22: >in the first session<

- Line 204: Write out this abbreviation at its first use (>CH<).

Response: We now revised all the three mistakes in the manuscript (see p. 1, line 6; p.2, line 26; p. 10, line 225)

19- The species studied is not mentioned in the abstract. Please make sure to update both the abstract in the article file and on the submission form.

Response: Thanks for your careful suggestion. we have added the description of species in the abstract (see p. 1, line 6) and submission form.

-------This ends our responses to all the comments-------
